# The Patella Pro study — effect of a knee brace on patellofemoral pain syndrome: design of a randomized clinical trial (DRKS-ID:DRKS00003291)

**DOI:** 10.1186/1471-2474-15-200

**Published:** 2014-06-10

**Authors:** Wolf Petersen, Andree Ellermann, Ingo Volker Rembitzki, Sven Scheffler, Mirco Herbort, Frederike Sophie Sprenker, Andrea Achtnich, Gert Peter Brüggemann, Raymond Best, Frank Hoffmann, Andreas Gösele Koppenburg, Christian Liebau

**Affiliations:** 1 Klinik für Orthopädie und Unfallchirurgie, Martin Luther Krankenhaus, Caspar Theyß Strasse 27-31, Berlin D-14193, Germany; 2 Arcus Sportklinik, Pforzheim, Germany; 3 Otto Bock, Duderstadt, Germany; 4 Asklepios, Harzkliniken GmbH, Fritz-König-Stift, Bad Harzburg, Germany; 5 Orthopädische Gemeinschaftspraxis, Berlin, Germany; 6 Klinik für Unfall-, Hand-, und Wiederherstellungschirurgie, Universitätsklinikum Münster, Münster, Germany; 7 Deutsche Sporthochschule Köln, Institut für Biomechanik, Köln, Germany; 8 Sportklinik Stuttgart, Stuttgart, Germany; 9 Orthopädische Klinik, Rosenheim, Germany; 10 Cross Klinik Basel, Olympic Medical Center, Basel, Switzerland

**Keywords:** Patellar maltracking, Dynamic valgus, Anterior knee pain, Functional malalignment, Chondromalacia patellae, Patellar orthosis, Patellar tape

## Abstract

**Background:**

Patellofemoral pain syndrome (PFPS) is a frequent cause of anterior knee pain predominantly affecting young female patients who do not have significant chondral damage. Development of PFPS is probably multifactorial, involving various knee, hip, and foot kinematic factors. Biomechanical studies have described patellar maltracking and dynamic valgus (functional malalignment) in patients with patellofemoral pain syndrome. The literature provides evidence for short-term use of nonsteroidal anti-inflammatory drugs; short-term medially directed taping; and exercise programs focusing on the lower extremity, hip, and trunk muscles. Evidence supporting the use of patellar braces is limited because previous studies have been low quality. The aim of this article is to publish the design of a prospective randomized trial that examines the outcomes of patients with PFPS after treatment with a new patellar brace (Patella Pro) that applies medially directed force on the patella.

**Methods/Design:**

For this multicenter trial, 156 patients (adolescents and young adults) with PFPS were recruited from orthopedic practices and orthopedic hospitals and randomly allocated to 3 months of supervised physiotherapy in combination with the Patella Pro brace or supervised physiotherapy alone. The primary outcome measures are pain (numerical analog scale); knee function (Kujala score and Knee Injury and Osteoarthritis Outcome Score); and self-reported perception of recovery at baseline, 6 weeks, 3 months, and 1 year.

**Discussion:**

Only limited evidence for the use of a patellar brace for the treatment of PFPS exists in the literature. Disputable evidence for the use of orthoses for PFPS patients has been presented in one meta-analysis, in which only one of three studies found the effect of a medially directed patellar brace to be significant. Because of these low-quality studies, the authors concluded that this evidence should be regarded as limited, and we feel there is a need for further well-designed studies to evaluate the effect of patellar bracing on PFPS-related pain. The Patella Pro study is a prospective randomized trial in which supervised physiotherapy in combination with a patellar brace is compared with supervised physiotherapy alone. This trial started in April 2012 and finished in October 2013.

**Trial registration:**

DRKS-ID:DRKS00003291, January 3^rd^, 2012

## Background

The incidence of anterior knee pain is high. Callaghan and Selfe conducted a literature search of English-language publications dated from January 2000 to December 2005 that showed the incidence of patellofemoral pain syndrome (PFPS) to be between 3% and 40% [1]. The authors found that evidence for the incidence of PFPS was taken almost entirely from source data in sports medicine or military settings [1]. Women are affected more than twice as often as men [2-4].

Patellofemoral pain syndrome (PFPS) is a common cause of anterior knee pain and mainly affects young women who do not have significant pathological changes in articular cartilage [3-9]. Therefore, PFPS is mainly a diagnosis of exclusion and, as such, demands careful clinical examination [5], which can detect such characteristics of PFPS as patellar maltracking [10].

A classic PFPS symptom is anterior knee pain that is provoked by prolonged sitting with bent knees, stair climbing, and sports activities [5-8]. Other associated manifestations include crepitus and functional deficit [5-8]. PFPS symptoms cause many athletes to limit their sports activities [2], and some authors have stated that PFPS can contribute to long-term patellofemoral osteoarthritis [11,12].

The role of patellar maltracking in the emergence of PFPS has long been a controversial issue. Recent studies, however, show that maltracking of the patella probably plays a key role [13-15]. There is evidence in the literature that the cause of patellar maltracking and subsequent imbalance of the vastus medialis and lateralis in some patients with PFPS may not be structural in nature; rather, dynamic or functional malalignment may underlie PFPS in these patients [6,7]. Functional or dynamic valgus may influence patellar tracking and lead to lateralization of the patella [14]. Recent research has shown that functional malalignment does not originate in the knee joint but results from internal rotation of the femur due to weakness of hip external rotators and abductors (gluteus medius and minimus) [16-19]. Further muscular imbalances involving the quadriceps and hamstrings can be found in these patients [20-22].

Muscular dysfunction probably plays a key role in the pathogenesis of PFPS; therefore, physiotherapy is the most frequently studied form of treatment [23,24], and there is strong evidence for the role of exercise in the treatment of PFPS in the literature. In a recent meta-analysis of 10 prospective randomized studies [23], each showed a positive effect of exercise on pain reduction. Positive results have been attributed to active stretching exercises, squats, cycle ergometry, isometric quadriceps exercises, active leg raises, leg press, and ascending and descending climbing exercises. Four of the exercise programs also included exercises to strengthen the hip abductors. In one study, exercises to stabilize the trunk, including the rectus abdominis, were analyzed. The most frequent duration of the exercise programs was 6 weeks, and exercises were performed two to four times daily.

Patellar braces are non-adhesive devices that, like taping, apply an external, medially directed force that may counteract lateral patellar maltracking. Draper et al. [13] have demonstrated by real-time MRI that a knee brace that applies a medially directed force on the patella can reduce patellar lateralization and tilt in women with PFPS significantly better than a bandage. Powers et al. [25] analyzed an orthosis that applied a medially directed force on the patella in PFPS patients and reported decreased pain and increased activation of the quadriceps. Selfe et al. [26,27] investigated the effect of patellar bracing and taping on the three-dimensional mechanics of the knee during a controlled step-down task in healthy and PFPS patients. These studies showed that bracing and taping offered coronal-plane and torsional control of the patella during eccentric contraction of the quadriceps in both PFPS patients and healthy subjects [26-29]. In addition to these biomechanical effects, Thijs et al. [30] showed that there was a significantly higher level of neuromotor and proprioceptive function with the application of the brace and sleeve, respectively, than without a brace or sleeve. Callaghan et al. [31] have shown that patellar taping modulates brain activity in several areas of the brain during a proprioceptive knee movement task. According to the meta-analysis published by D'hondt et al. [32], the use of a patellar brace has positive effects on pain, function (Kjuala score), and patellofemoral congruence angle compared with an untreated control group. However, because of the low quality of the studies, the authors concluded that this evidence should be regarded as limited. Warden et al. [33] also found disputable evidence for the use of orthoses in PFPS patients. In this meta-analysis, only one of three studies reported a significant effect with a medially directed patella brace, whereas in the other two studies the effect was not significant. In these studies no difference was found between medially directed bracing and sham bracing.

Therefore, we conclude that there is a need for further well-designed studies to evaluate the effect of patellar bracing on pain in PFPS. The Patella Pro (Otto Bock, Duderstadt, Germany) is a new knee brace designed to apply a medially directed force on the patella to counteract lateral maltracking. The main characteristic of this patella brace is the dynamic tracking system. The risk of improper tracking of the patella is particularly high for flexion angles between 0 and 30 degrees, when the patella is not guided by the patellofemoral groove. The tracking system of the Patella Pro was designed with a sleeve that can apply a dynamic medially directed force on the patella and track the patella within this range of motion. The pressure from the tracking system decreases with increasing flexion angle [34], thus protecting the patella from pressure where it is not needed. The ability of this brace to counteract lateral patellar maltracking, thus improving biomechanical function, was demonstrated in a biomechanical cadaver study at the German Sports University in Cologne [28], which showed that the Patella Pro has the potential to medialize the patella during the entire range of motion.

The Patella Pro clinical trial will target patients 18–50 years of age presenting with symptoms of PFPS. The details of the study protocol are presented here. Our hypothesis is that there is a synergistic effect between the Patella Pro brace and exercise.

## Methods/Design

### Study design

**Figure 1:**
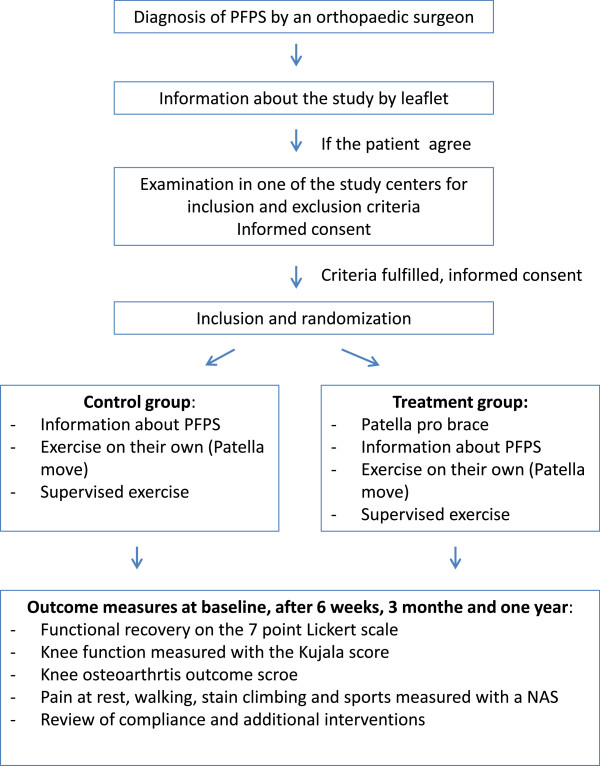
Flow chart of the study design.

This study is a randomized clinical trial examining the short-term effectiveness of a patellar brace (Patella Pro, Otto Bock, Duderstadt, Germany) in combination with exercise and information on the mechanism of PFPS, compared with exercise and information on the mechanism of PFPS only (Figure 1). The study design is based on the protocol of an intervention study for the treatment of PFPS with exercise [29].

This study design was approved by the medical ethics committee of the medical faculty of the Charité—Universitätsmedizin Berlin. All patients provided written informed consent.

The study protocol is registered with the *Deutsches Register Klinischer Studien* (“German Clinical Trials Register”) as DRKS-ID number DRKS00003291.

### Study centers

This study is a multicenter clinical trial. Patients were recruited from the following hospitals and orthopedic practices:

1. Klinik für Orthopädie und Unfallchirurgie, Martin Luther Krankenhaus, Berlin Grunewald, Germany

2. Arcus Sportklinik, Pforzheim, Germany

3. Asklepios, Harzkliniken GmbH, Fritz-König-Stift, Bad Harzburg, Germany

4. Orthopädische Gemeinschaftspraxis, Berlin, Germany

5. Klinik für Unfall-, Hand-, und Wiederherstellungschirurgie, Universitätsklinikum Münster, Germany

6. Orthopädische Klinik, Rosenheim, Germany.

The clinical outcome parameters will be collected and analyzed at the Klinik für Orthopädie und Unfallchirurgie, Martin Luther Krankenhaus, Berlin Grunewald, Germany.

### Patient selection

Adults aged 18–50 years with PFPS symptoms lasting longer than 2 months but not longer than 2 years are eligible to participate. The recruitment period took place from April 2012 to October 2013. The first results of the study will be available in 2014.

### Inclusion and exclusion criteria

Inclusion criteria are age between 18 and 50 years and the presence of three of the following symptoms lasting longer than 2 months but not longer than 2 years: Anterior knee pain when running, climbing stairs, cycling, sitting with a bent knee, or performing squats. Pain level is not an inclusion criterion.

Exclusion criteria include the following: Kellgren-Lawrence grade 3 to grade 4 osteoarthritis [35]; local grade 3 to grade 4 cartilage damage on MRI, measured using the Gluckert grading system [36]; subluxation of the patella; previous knee injury (cruciate ligaments); tendinosis of the patellar tendon; Osgood-Schlatter disease; osteochondritis dissecans; valgus with more than 3 fingerbreadths of intermalleolar distance; and varus with more than 2 fingerbreadths of intercondylar distance.

Diagnoses are made on the basis of medical history, clinical findings, and existing x-ray and magnetic resonance imaging (MRI) scans. Patients without existing MRI and x-ray were not recruited.

## Informed consent

Patients who qualify as study participants on the basis of inclusion and exclusion criteria are informed about the study. This information includes written general information on patellofemoral pain syndrome and a written description of the present study. These instructions include information about the purpose of the study and potential risks. All participants must sign an informed-consent form.

### Sample size calculation

In an intervention study by Clark et al. [37], the difference in recovery rates between the intervention and control groups was 22%. This difference was statistically significant (power 0.8, alpha 0.05). With a potential dropout rate of about 5-10%, approximately 144 patients (135 patients +10 patients) must be enrolled in this study (power 0.80, alpha 0.05).

### Intervention

The two treatment groups in this trial undergo the following interventions:

Group 1: Patients receive written information about the program, including schematic drawings of the five PFPS exercises, which they perform on their own (home exercise) in a structured program (Patella Move). The five exercises are as follows: Sitting and flexing the knee, sitting and tensing the quadriceps, two-legged stance and squat, one-legged stance and squat, and one-legged stance and lateral pressure. Patients are instructed to complete the three sets of six repetitions per leg over the course of the day and to take a break the following day. The duration of the Patella Move program is not limited. The Patella Move program involves gradually increasing intensity to a maximum that is determined individually on the basis of actual symptoms [34]. The instruction sheet tells patients: “Do not stress yourself” and “If you feel insecure, leave out an exercise”.

**Figure 2:**
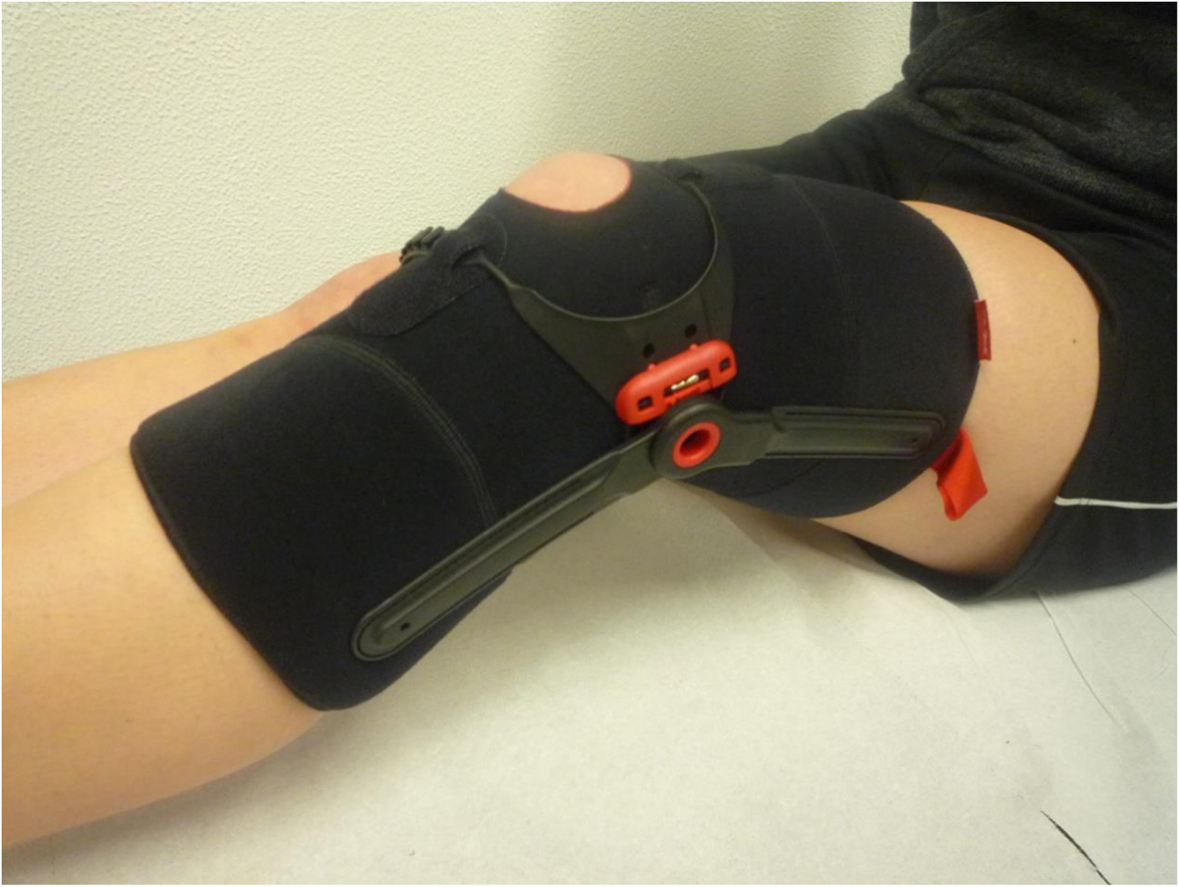
**Patellar brace (Patella Pro, Otto Bock, Duderstadt, Germany).** The sleeve of the brace can apply a medially directed force on the patella. Consent from the patient for this image to be published was obtained.

Supervised exercises: Study participants receive a *Krankengymnastik am Gerät* (“prescription for physical therapy using the device”). In Germany, *Krankengymnastik am Gerät* is regulated by law (§ 125 Abs. 1 SGB V vom1. August 2001 in der Fassung vom 1. Juni 2006) [38]. The diagnosis *patellofemoral pain syndrome* is noted on the prescription. This prescription ensures a structured training program using the following exercises or devices: Functional leg press, treadmill, ergometer, stepper, angle table, and vertical pull apparatus. After a detailed analysis, the physical therapist creates a customized training plan for each patient [38]. The goal of *Krankengymnastik am Gerät* is to improve strength, coordination, endurance, and flexibility of the lower extremity, including the hip muscles (38). The duration of one session is 60 minutes (38). The prescription is accompanied by detailed instructions and information about the study for the physiotherapist. Although patients in group 1 will not be wearing the Patella Pro device, this approach ensures that the training program designed for this diagnosis at all study centers in Germany is largely homogeneous. The duration of the supervised exercise program is 6 weeks (12 units). The costs of physiotherapy are covered by Otto Bock.Group 2: Patients receive detailed information about PFPS; written information about the program and instructions on Patella Move exercises, which they perform as a structured home-exercise program; and supervised physiotherapy, all as described for group 1. However, in this group, study participants also receive a patellar brace (Patella Pro) (Figure 2, consent from the patient for this image to be published was obtained). Medially directed force is applied to the patella by a tracking system. The brace is adapted to the patient by the study physician at the study center, and its function is explained to the patient. Study participants also receive appropriate patient information about the Patella pro brace provided by Otto Bock. They are instructed to use the orthosis over a period of 6 weeks for at least 6 hours a day.

### Co-interventions

During the 1-year course of the study, the following co-interventions are allowed: Application of ice, ointment dressings, and consumption of oral analgesics (nonsteroidal anti-inflammatory drugs or paracetamol). Patients are asked to report on co-interventions after 6 weeks and 3 months and during the 1-year follow-up.

### Randomization

After patients are recruited and informed consent has been obtained, the patients are randomized to the two treatment groups. All study centers receive the randomization documents in a sealed envelope. After opening the envelope, the group to which each patient is assigned can be seen. Patients are allocated to either group 1 or group 2.

### Baseline examination

The following data are collected during the baseline examination: Date; patient age, sex, and contact information; left or right knee, symptoms, and current treatment; medical history; clinical examination findings, including range of motion, stability tests (Lachman, pivot-shift, and posterior drawer tests); meniscus signs; intra-articular effusion; soft-tissue swelling; circumference measurements; and leg axis, including intercondylar and intermalleolar distances.

### Data processing

Patient name and contact information, along with the results of the baseline examination, are transmitted to the evaluation center (Martin Luther Hospital, Berlin). After evaluation, the data are processed anonymously. Any publication of the data is also done anonymously.

### Outcome parameters

Primary outcome measures are subjective assessments of recovery using a seven-point Likert scale [29,37] at 3 months and 1 year. This parameter was used for the sample size calculation.

Secondary outcome measures are subjective assessment of recovery using a seven-point Likert scale at 6 weeks; modified functional Kujala score [39] without the parameters muscular atrophy and flexion; the German version of the Knee Injury and Osteoarthritis Outcome Score (KOOS) [40,41]; pain at rest and with walking, stair climbing, sitting and sports activity, reported on a numerical scale (0 to 100); and review of compliance and additional interventions. All these measurements are evaluated via questionnaire at baseline, 6 weeks, 3 months and 1 year.

The seven-point (*completely recovered, strongly recovered, significant improvement, moderate improvement, little improvement, slightly recovered, worse than ever*) Likert scale used in the present study was used previously by Van Linschoten et al. [29] to study the effect of supervised physiotherapy on PFPS patients (Table 1). Patients were deemed to have recovered if they rated themselves *fully recovered* or *strongly recovered*, whereas those who rated themselves *slightly recovered* to *worse than ever* were deemed not to have recovered. This threshold was used to dichotomize perceived recovery into two clear categories: “recovered” and “not recovered”.

**Table 1:** Seven-point Likert scale used to measure recovery

	**1**	**2**	**3**	**4**	**5**	**6**	**7**
**German (original version)**	Komplette Heilung	Fast geheilt	Deutliche Besserung	Mäßige Besserung	Wenig Besserung	Geringfügig gebessert	So schlecht wie bisher
**English translation**	Completely recovered	Strongly recovered	Significant improvement	Moderate improvement	Little improvement	Slightly recovered	Worse than ever

Patients were classified to have recovered if they rated themselves *fully recovered* or *strongly recovered.* Those who rated themselves as *slightly recovered* to *worse than ever* were classified as not recovered.

The Kujala score is a disease-specific, validated disability scale for patellofemoral disorders that ranges from 0 (complete disability) to 100 (fully functional) [39].

The validated German version of the KOOS is self-explanatory and consists of five subscales; pain, symptoms, sports/recreational activities, activities of daily living, and function [40]. Standardized answer options are given (five Likert boxes), and each question is assigned a score from 0 to 4. A normalized score (100 indicating no symptoms and 0 indicating extreme symptoms) is calculated for each subscale [40,41]. A total score has not been validated and is not recommended [40].

Pain at rest and with walking, stair climbing, sitting, and sports activity is marked by the patient on a numerical scale (0 to 100).

All patients are asked during telephone interviews at 6, 12, and 54 weeks if they have adhered to treatment. It is noted if they have not continued using the brace, performed the home-exercise program, or attended supervised physiotherapy sessions.

### Expected results

In the study of Clark et al. [37], functional recovery was significantly higher in a group of PFPS patients who performed supervised physiotherapy. We hypothesize that the passive realignment provided by the Patella Pro brace decreases pain during exercises. Therefore, we expect that the exercises in group 2 are more effective than in group 1. This should result in a significantly higher rate of recovery in group 2.

Van Linschoten et al. [29] observed a decrease in pain at rest and with activity as well as an increase in function after 3 months in a group receiving supervised physiotherapy. Therefore, decreased pain and increased function are also expected in all patients in the present study. The hypothesis of this study is that patients using the brace will experience greater improvements (higher scores) in the KOOS subscales and Kujala score and greater reductions in pain than non-braced patients.

### Statistical analyses

To evaluate additional effects of the patellar brace on supervised exercise and home exercise in patients with PFPS, between-group differences in clinical outcomes will be analyzed on the basis of intention to treat. Logistic regression techniques are used for dichotomous outcome parameters. Continuous outcomes will be analyzed with linear regression techniques. Recovery, as measured by the seven-point Likert scale, will be dichotomized to recovered (fully or strongly) or not recovered (slightly to strongly worsened). Other outcome measures will be analyzed as continuous variables.

Statistical analyses are performed at Medistat, Kiel, Germany.

## Discussion

The Patella Pro study was designed after reviewing the literature on therapy for patients with PFPS [9,29]. The PEX study protocol published by Van Linschoten et al. [9] served as the model for this trial because it is a high-quality protocol for a trial examining an intervention for patients with a PFPS.

Treatment options for PFPS patients are patellar taping and bracing [7]. The exact mechanism of action of both modalities is unknown. Some authors have been able to demonstrate biomechanical effects of taping and brace application; others have demonstrated improved proprioception and effects on brain activity [30,31]. A meta-analysis of studies limited in number and quality has shown that brace therapy may have a positive effect on PFPS [33]. Conflicting results of studies of the effects of patellar bracing [33] could also be the result of the brace design. The present study will contribute to the elucidation of this research question. We expect an answer about the clinical effect of the Patellar pro brace for the treatment of PFPS. Unfortunately we cannot answer if the effect is due to the medially directed force or a proprioceptive effect.

Based on the available literature regarding tape application in PFPS patients [33,42,43], our research group expects to discover benefits of brace treatment, including perceived recovery and improvements in pain severity and functional disability, in the Patella Pro study.

A limitation of the present study design is that only patients with an existing MRI and x-ray are included. This could be a selection bias, as only those patients with greater attention to their pathological condition are selected. Another limitation could be the age band going up to 50 as this group may have other symptoms. However, the exclusion criteria will mediate against some of these.

## Competing interests

The study sponsor is Otto Bock, Duderstadt, Germany. WP, AE, FH, CL, RB, GB, and AG are consultants to Otto Bock. IR is an employee of Otto Bock. SS and MH receive funding from Otto Bock for inclusion of patients.

## Authors' contributions

AA, SS, MH, AE, CL, RB, GB, FH, IR, and AG developed the design of this trial and contributed to the content of the article. All these authors are also responsible for patient recruitment. WP participated in the design and coordination of the study and wrote the article. FS coordinated the trial and is responsible for data acquisition. All authors read and approved the final article.

## Authors’ information

WP: Prof. Dr. Wolf Petersen, chief physician at the orthopedic and trauma department of the Martin Luther Hospital, Berlin, and associate editor of the journal *Arthroscopy*. AE: Dr. Andre Ellermann, chief physician at the Arcus Sports Clinic, Pforzheim.

IR: Ingo Rembitzki, physiotherapist, employee of Otto Bock, Duderstadt. SS: PD Dr. Sven Scheffler, orthopedic surgeon in a private practice in Berlin. MH: Dr. Mirco Herbort, orthopedic surgeon at the department of trauma-, hand-, and reconstructive surgery at the Westfalian Wilhelms University in Münster. FS: Frederike Sprenker, study nurse at the orthopedic and trauma department of the Martin Luther Hospital, Berlin and medical student at the Freie Universität Berlin. AA: Dr. Andrea Achtnich, resident at the orthopedic and trauma department of the Martin Luther Hospital, Berlin. PB: Prof. Gerd Peter Brüggemann, director of the biomechanical institute, Deutsche Sport Hochschule, Köln. RB: Dr. Raymond Best, orthopedic surgeon at the Sportklinik Stuttgard, team physician of the first league soccer team VFB Stuttgard. FH: Dr. Frank Hoffmann, chief physician at the department of orthopedic surgery, Klinikum, Rosenheim. AG: Dr. Andreas Gösele Koppenburg, orthopedic surgeon at the cross clinic Basel, team physician, Leopard/Trek cycling team. CL: Dr. Christian Liebau, chief physician at the department of orthopedic surgery, Askleps Klinik, Bad Harzburg.

## Pre-publication history

The pre-publication history for this paper can be accessed here:

http://www.biomedcentral.com/1471-2474/15/200/prepub
